# Type 2 diabetes mellitus-associated transcriptome alterations in cortical neurones and associated neurovascular unit cells in the ageing brain

**DOI:** 10.1186/s40478-020-01109-y

**Published:** 2021-01-06

**Authors:** Joanna J. Bury, Annabelle Chambers, Paul R. Heath, Paul G. Ince, Pamela J. Shaw, Fiona E. Matthews, Carol Brayne, Julie E. Simpson, Stephen B. Wharton, the Cognitive Function and Ageing Study

**Affiliations:** 1 Sheffield Institute for Translational Neuroscience, University of Sheffield, 385a Glossop Road, Sheffield, S10 2HQ UK; 2 Population Health Sciences Institute, Newcastle University, Newcastle upon Tyne, UK; 3 Institute of Public Health, University of Cambridge, Cambridge, UK

**Keywords:** Ageing, Cortical neurone, Dementia, Differential expression, Neurovascular unit, Transcriptome, Type 2 diabetes mellitus

## Abstract

Type 2 diabetes mellitus (T2D), characterised by peripheral insulin resistance, is a risk factor for dementia. In addition to its contribution to small and large vessel disease, T2D may directly damage cells of the brain neurovascular unit. In this study, we investigated the transcriptomic changes in cortical neurones, and associated astrocytes and endothelial cells of the neurovascular unit, in the ageing brain. Neurone, astrocyte, and endothelial cell-enriched mRNA, obtained by immuno-laser capture microdissection of temporal cortex (Brodmann area 21/22) from 6 cases with self-reported T2D in the Cognitive Function and Ageing Study neuropathology cohort, and an equal number of age and sex-matched controls, was assessed by microarray analysis. Integrated Molecular Pathway Level Analysis was performed using the Kyoto Encyclopaedia of Genes and Genomes database on significantly differentially expressed genes, defined as *P* < 0.05 and fold-change ± 1.2. Hub genes identified from Weighted Gene Co-expression Network Analysis were validated in neurones using the NanoString nCounter platform. The expression and cellular localisation of proteins encoded by selected candidate genes were confirmed by immunohistochemistry. 912, 2202, and 1227 genes were significantly differentially expressed between cases with self-reported T2D and controls in neurones, astrocytes, and endothelial cells respectively. Changes in cortical neurones included alterations in insulin and other signalling pathways, cell cycle, cellular senescence, inflammatory mediators, and components of the mitochondrial respiratory electron transport chain. Impaired insulin signalling was shared by neurovascular unit cells with, additionally, apoptotic pathway changes in astrocytes and dysregulation of advanced glycation end-product signalling in endothelial cells. Transcriptomic analysis identified changes in key cellular pathways associated with T2D that may contribute to neuronal damage and dysfunction. These effects on brain cells potentially contribute to a diabetic dementia, and may provide novel approaches for therapeutic intervention.

## Introduction

Type 2 diabetes mellitus (T2D), characterised by peripheral insulin resistance (IR), is an increasing problem in affluent societies. T2D increases with age and is associated with multiple organ complications. It is related to metabolic syndrome (MS), which includes central obesity, hypertension, hyperglycaemia, raised serum triglycerides, and lowered serum levels of high-density lipoprotein [1].

T2D/MS is an important risk factor for dementia [55]. T2D/MS is also a risk factor for vascular dementia (VaD) and Alzheimer’s disease (AD), although the evidence for the latter is less clear [11, 60], and T2D does not appear to be associated with increased AD neuropathological changes [4, 33]. Importantly, whereas the major causes of dementia are currently untreatable, some cases of dementia may be accounted for by risk factors that can be modified. Work on the United Kingdom population-based Cognitive Function and Ageing Study (CFAS) neuropathology cohort suggests that up to a third of AD cases are attributable to modifiable risk factors, including T2D/MS and midlife obesity, with attributable risks for AD in the United Kingdom of 1.9% and 6.6%, respectively [43]. However, a recent systematic review did not find that various treatment strategies for T2D, aimed at systemic glycaemic control, affect cognitive outcome [47].

The brain neurovascular unit (NVU), comprising cerebral endothelium, pericytes, astrocytes, and neurones, is an important functional unit involved in homeostasis, through regulation of the blood–brain barrier, and neuronal function through astrocytic neuronal support and neurovascular coupling [23]. Changes to the NVU are of increasing importance in neurodegeneration [71], and in vivo imaging studies have shown that alterations to neurovascular coupling occur early in T2D [15]. In addition to its contribution to small and large vessel disease, T2D may affect the brain NVU through a number of candidate mechanisms. T2D/MS is associated with systemic metabolic abnormalities [16, 35], and endothelial pathology in the periphery can result from inflammation, oxidative stress, hyperglycaemia, and signalling abnormalities [46]. The hyperglycaemia of T2D/MS can result in carbonyl stress, causing glycation with the formation of advanced glycation end-products (AGEs), which may interact with receptors for AGEs (RAGEs), and cause inflammation and oxidative stress [54, 61]. AGE formation may also increase beta-amyloid (Aβ) pathology and tau phosphorylation [3]. IR is a characteristic feature of T2D/MS. There appears to be a relationship between peripheral and central IR [69]. Neuronal IR has also been found in AD [37, 57], and reduced insulin signalling can increase tau phosphorylation in experimental models [10]. However, Aβ molecular pathology may also affect insulin signalling [56, 70]. Amylin (islet amyloid polypeptide), which accumulates in pancreatic islets in T2D/MS, may accumulate in the brain and contribute to AD pathogenesis [5].

It is possible, therefore, that T2D/MS directly damages cells of the brain NVU, in addition to its effects on arteries and microvasculature. Whilst changes to neurones have long been considered central to dementia pathology, astrocyte cellular pathology is also recognised as being important [20], whilst endothelial cells and microvascular function are thought to be affected early in AD and to contribute to its development [12, 25, 42].

However, these potential mechanisms of brain cell damage in T2D/MS remain poorly understood. We therefore investigated the transcriptomic changes in cortical neurones in donated brains from individuals with self-reported T2D compared to those without. We also investigated the concurrent changes in astrocytes and endothelial cells, to set the neuronal changes in the context of the brain NVU. We used donated brain tissue from the CFAS neuropathology cohort. CFAS is a longitudinal study of cognitive impairment and frailty in the over-65 age group, with a population-representative neuropathology cohort [8, 36, 66]. We identified changes in key cellular pathways that might contribute to neuronal damage and dysfunction, potentially contributing to a diabetic dementia.

## Materials and methods

### Human central nervous system tissue

Post-mortem lateral temporal cortex (Brodmann area 21/22) was obtained from the CFAS neuropathology cohort (www.cfas.ac.uk) [66], in accordance with Research Ethics Committee approval (15/SW/0246). Six cases with self-reported T2D, a group size considered adequate for gene expression studies [44, 59], and an equal number of age and sex-matched controls were selected to have minimal AD neuropathology, as defined by low Braak neurofibrillary tangle stage (stage 0–II) [6], without confounding pathologies (Table 1). Dementia status at death was assessed using an algorithmic approach, as previously described [66]; this was not used for case selection. A tissue pH cut-off of 6.0 was used, consistent with previous studies [38, 58]. The mean brain pH was 6.6 (standard deviation [SD] 0.4; range 6.1–7.3). The median post-mortem interval (PMI) was 32.3 h (interquartile range 16–36 h). The average age at death was 79.3 years (SD 7.2 years; range 73–92 years).

**Table 1:** Demographic, pathological and clinical data of CFAS brain donors

Case	Age (y)	Gender	PMI (h)	Brain pH	Braak stage	CERAD^a^	Thal Phase	CAA	Dementia^1^at death	Cause of death^2^
C1	87	F	60	6.1	II	None	1	No	No	Depressive episode, unspecified
C2	92	M	33	6.8	II	None	2	Yes	Yes	Chronic ischaemic heart disease
C3	74	M	N/A	6.3	II	Moderate	2	No	No	Acute myocardial infarction
C4	75	M	29	7.3	0	Mild	3	Yes	No	Acute myocardial infarction
C5	73	F	20	6.5	0	None	0	No	No	Amyloidosis
C6	78	F	17	6.9	I	None	1	No	No	Pulmonary embolism
D1	73	M	104	6.2	II	None	0	Yes	No	Cellulitis, unspecified
D2	76	M	16	6.6	II	Moderate	2	Yes	No	Chronic ischaemic heart disease
D3	75	F	5	6.3	I	None	2	No	No	Acute myocardial infarction
D4	73	M	23	6.9	I	None	1	Yes	No	Interstitial pulmonary disease with fibrosis
D5	84	F	12	7.3	II	None	3	Yes	No	Chronic ischaemic heart disease
D6	91	M	36	6.4	II	None	0	Yes	No	Unspecified diabetes mellitus without complications

C, control; CFAS, Cognitive Function and Ageing Study; D, diabetic (type 2 diabetes mellitus); F, female; h, hour; M, male; PMI, post-mortem interval; y, year; CAA, cerebral amyloid angiopathy present or absent

^a^CERAD maximum neuritic plaque score

^1^Dementia status at death

^2^Cause of death as recorded from death certification data

### Immuno-laser capture microdissection

Neurones, astrocytes, and capillary endothelial cells were isolated from separate sections of the same frozen blocks of lateral temporal cortex, using rapid immuno-laser capture microdissection (LCM), which preserves RNA quality sufficiently for array analysis, as described previously [53, 63]. This method produces cell-type enriched, but not pure, samples. Toluidine blue-stained neurones, glial fibrillary acidic protein (GFAP)-stained astrocytes, and collagen IV-stained endothelial cells were isolated from each case using the PixCell II LCM System (Arcturus Engineering, Mountain View, CA, USA) (Table 2). Total RNA was extracted using PicoPure^®^ RNA Isolation Kits (Arcturus BioScience, Mountain View, CA, USA), according to the manufacturer’s protocol, with typical yields of 86.0 ng (SD 2.5 ng; range 52.3–133.7 ng), 148.4 ng (SD 2.8 ng; range 101.0–186.8 ng), and 141.2 ng (SD 8.0 ng; range 31.6–311.4 ng) of total RNA from neurones, astrocytes, and endothelial cells, respectively. The concentration and purity (*A*_260_/*A*_280_ ratio) of the total RNA were measured using the NanoDrop 1000 Spectrophotometer (Thermo Scientific, Wilmington, DE, USA). The quality of the total RNA was assessed using the 2100 Bioanalyser with RNA 6000 Pico LabChip Kits (Agilent, Palo Alto, CA, USA).

**Table 2:** Antibody source and specificity

Antibody	Isotype	Dilution (time, temp)	Antigen retrieval	Supplier
AGE	Rabbit IgG	1:2000 (60 min, RT)	MW 10 min, TSC	Abcam, UK
COL4	Rabbit IgG	1:200 (3 min, RT)	N/A	Abcam, UK
COX5b	Rabbit IgG	1:50 (60 min, RT)	MW 10 min, EDTA	Thermo Fisher Scientific, UK
FOXO3a	Rabbit IgG	1:100 (O/N, 4 °C)	PC, TSC	Abcam, UK
GFAP	Rabbit IgG	1:50 (3 min, RT)	N/A	Dako
γH2AX	Rabbit IgG	1:2000 (60 min, RT)	PC, EDTA	R&D Systems, UK
IGF-1Rβ	Rabbit IgG	1:25 (60 min, RT)	N/A	Santa Cruz Biotechnology, UK
NDUFb6	Rabbit IgG	1:100 (60 min, RT)	MW 10 min, EDTA	Sigma-Aldrich, UK
p53	Mouse IgG	1:50 (O/N, 4 °C)	PC, MenaPath Access Super	Santa Cruz Biotechnology, UK
TGFβ1	Rabbit IgG	1:400 (60 min, RT)	PC, EDTA	Abcam, UK

AGE, advanced glycation end-product; COL4, collagen IV; COX5b, cytochrome C oxidase subunit 5B; EDTA, ethylenediaminetetraacetic acid; FOXO3a, forkhead box O3; GFAP, glial fibrillary acid protein; IGF-1Rβ, insulin-like growth factor 1 receptor beta; IgG, immunoglobulin G; MW, microwave; N/A, not applicable (frozen tissue); NDUFb6, NADH:ubiquinone oxidoreductase subunit B6; O/N, overnight; p53, tumour protein p53; PC, pressure cooker; RT, room temperature; TB, toluidine blue; TGFβ1, transforming growth factor beta 1; TSC, trisodium citrate; γH2AX, gamma H2A histone family member X

### RNA amplification and microarray hybridisation

Linear amplification of total RNA was performed following Eberwine’s procedure [62], using GeneChip™ 3′ IVT Pico Kits (Applied Biosystems, Warrington, UK). Total RNA (10.0 ng) was reverse-transcribed to synthesise the first-strand of complementary DNA (cDNA), using primers containing a T7 promoter sequence. Single-stranded cDNA was converted into double-stranded cDNA, using DNA polymerase and RNase H, to simultaneously degrade the RNA and synthesise the second-strand of cDNA. Double-stranded cDNA was used as the template for an overnight in vitro transcription (IVT) reaction to synthesise anti-sense RNA (complementary RNA [cRNA]), using T7 RNA polymerase. In the second cycle of cDNA synthesis, random primers were used to reverse-transcribe the cRNA, to obtain sense-strand cDNA. The quality of the cRNA and double-stranded cDNA were assessed using the 2100 Bioanalyser with RNA 6000 Pico LabChip Kits (Agilent, Palo Alto, CA, USA).

According to the manufacturer’s instructions, 6.6 µg of purified double-stranded cDNA was fragmented using uracil-DNA glycosylase (UDG) and apurinic/apyrimidinic endonuclease 1 (APE1), and biotin-labelled with terminal deoxynucleotidyl transferase (TdT), using the Affymetrix proprietary DNA Labelling Reagent. The labelled cDNA was hybridised onto Human Genome U133 Plus 2.0 GeneChip^Ⓡ^ Arrays (16 h at 45 °C, with rotation at 60 rpm) (Affymetrix UK, High Wycombe, UK), containing 54,000 probe sets, coding for > 47,000 transcripts and variants, including 38,500 unique human genes.

Post-hybridisation washing and staining were performed using the Affymetrix Fluidics Station 450 and Affymetrix GeneChip^Ⓡ^ Operating Software (GCOS). The arrays were scanned using the Affymetrix GeneChip^Ⓡ^ 3000 7G Scanner. Hybridisation quality was determined using Affymetrix Expression Console™ version 1.4.1.46 (Affymetrix, Inc., Santa Clara, CA, USA).

One control, C6 (Table 1), yielded insufficient quantities of double-stranded cDNA for microarray hybridisation. The sample was repeated. It failed the amplification step twice and was excluded from downstream analysis.

### Microarray analysis

Raw signal intensity (.CEL) files from 6 cases with self-reported T2D and 5 age- and sex-matched controls were imported into Qlucore Omics Explorer version 3.4 (Qlucore AB, Lund, Sweden). Normalisation was performed using the Robust Multi-array Average (RMA) algorithm [24, 34]. Two-group comparisons were conducted using a two-tailed, unpaired Student’s *t* test, without performing a correction for multiple testing. Significantly differentially expressed genes (DEGs) were identified using an unadjusted *P* < 0.05 and a fold-change (FC) threshold of plus or minus 1.2. Comparisons were drawn using jVenn: an interactive Venn diagram viewer (http://jvenn.toulouse.inra.fr) [2].

### Integrated molecular pathway level analysis

A Wilcoxon pathway enrichment analysis, based on gene symbol and FC, was performed for each cell type using Integrated Molecular Pathway Level Analysis (IMPaLA) version 11, build April 2018 (http://impala.molgen.mpg.de/) [27]. For genes with multiple probe sets, the mean FC was used. Significantly enriched pathways in the Kyoto Encyclopaedia of Genes and Genomes (KEGG) database (https://www.genome.jp/kegg/) [28, 29] were identified using an unadjusted *P* < 0.05 (Additional file 4: Table S1).

### Weighted gene co-expression network analysis

Weighted gene co-expression network analysis (WGCNA) was performed using the GeneMANIA plugin [39] for Cytoscape version 3.7.2 (Cytoscape Consortium, San Diego, CA, USA) [49]. Networks were created using the IMPaLA-generated lists of DEGs enriched in KEGG pathways associated with (1) Diabetes and dementia (*n *= 73, *P *< 0.05) (Additional file 1: Fig. S1), (2) Diabetic complications (*n *= 101, *P* < 0.05) (Additional file 2: Fig. S2), (3) DNA damage response (*n *= 87, *P *< 0.05) (Additional file 3: Fig. S3a), and (4) Autophagy (*n *= 31, *P *< 0.001) (Additional file 3: Fig. S3b). The Diabetes and dementia network included insulin signalling, AGE-RAGE signalling in diabetic complications, and Alzheimer disease. The Diabetic complications network included AGE-RAGE signalling in diabetic complications, chemokine signalling (inflammation), hypoxia inducible factor-1 (HIF-1) signalling, fluid shear stress and atherosclerosis, and non-alcoholic fatty liver disease (NAFLD). The DNA damage response network included cell cycle, cellular senescence, p53 signalling, apoptosis, and necroptosis (inflammatory cell death). The brain NVU was analysed as a whole. Network weighting was assigned based on query genes, so as to maximise connectivity among input genes. The maximum number of related genes or attributes was zero [18, 65]. Two gene products are linked in red, if they participated in the same reaction within a pathway [67]. The strengths of relationships are represented by the intensity of the colour and the thickness of the interconnecting lines.

### Microarray validation in neurones: NanoString nCounter platform

Hub genes identified through WGCNA were validated using NanoString nCounter XT CodeSet Gene Expression Assays (NanoString Technologies, Seattle, WA, USA). A custom CodeSet (C6959X1) was designed in consultation with the NanoString Bioinformatics team and manufactured by NanoString Technologies (Additional file 5: Table S2). The CodeSet was created by combining 69 probe sets from NanoString’s commercially available nCounter Human Neuropathology and nCounter Human Inflammation panels. Actin, beta (*ACTB*), glyceraldehyde-3-phosphate dehydrogenase (*GAPDH*), and ribosomal protein, large, P0 (*RPLP0*) were selected as housekeeping genes, based on the stability of their expression in the microarray study. Twenty-eight genes were included from the Diabetes and dementia network (Additional file 1: Fig. S1), 42 from the Diabetic complications network (Additional file 2: Fig. S2), 37 from the DNA damage response network (Additional file 3: Fig. S3a), and 11 from the Autophagy network (Additional file 3: Fig. S3b), with 32 genes common to two or more networks. The nCounter Analysis System uses molecular ‘barcodes’ and single-molecule imaging to detect and count hundreds of unique transcripts in a single reaction. Each colour-coded barcode is covalently attached to a single target-specific probe corresponding to a gene of interest. Mixed together with controls, they form a multiplexed CodeSet [21, 22, 32].

Total RNA, approximately 165.0 ng (SD 23.3 ng; range 126.5–202.8 ng), was concentrated to a volume of 5.0 µL, using a miVac DNA concentrator (GeneVac Ltd., Suffolk, UK), with the temperature set at 31 °C. After an overnight hybridisation (16 h at 65 °C in a thermocycler) with the target-specific biotinylated capture probes and barcode-containing reporter probes in solution, excess probes were removed, and target-probe complexes immobilised and aligned in the nCounter cartridge, which was loaded into the nCounter *SPRINT™* Profiler (NanoString Technologies, Seattle, WA, USA) for image acquisition and data processing. Gene expression was measured by counting the number of times the colour-coded barcode for each gene was detected.

### NanoString analysis of neurones

Reporter Code Count (RCC) files were imported into nSolver™ Analysis Software 4.0 (NanoString Technologies, Seattle, WA, USA). The manufacturer’s recommended default parameters for quality control flagging were used for imaging (field of view registration, < 75%), binding density (< 0.1 or > 2.25), positive control linearity (*R*^2^ value < 0.95), and positive control limit of detection (0.5 fM positive control ≤ 2 SD above the mean of the negative controls). The background threshold was calculated using the geometric mean of the negative controls. Each sample was first normalised to the geometric mean of the positive controls (with default flagging of normalisation factors, < 0.3 and > 3), followed by normalisation to the geometric mean of the housekeeping genes, *ACTB*, *GAPDH*, and *RPLP0* (with default flagging of normalisation factors, < 0.1 and > 10). Differential expression was evaluated using the nCounter Advanced Analysis plugin version 2.0.115 (NanoString Technologies, Seattle, WA, USA). Statistical significance was determined using an unadjusted *P* < 0.05.

### Immunohistochemical validation of protein expression

To confirm the expression and cellular localisation of proteins encoded by selected candidate genes, immunohistochemical staining of sections of lateral temporal cortex from the same cases as the microarray cohort was performed, using a standard horseradish peroxidase-conjugated avidin–biotin complex method, using Vectastain Elite Kits (Vector Laboratories, Peterborough, UK). Peroxidase activity was detected using diaminobenzidine (Vector Laboratories, Peterborough, UK). The primary antibodies used and their experimental conditions are summarised in Table 2.

## Results

### Enrichment of neuronal, astrocytic, and endothelial cell populations

Neurofilament protein, light chain (*NEFL*) and neurofilament protein, heavy chain (*NEFH*) were highly expressed in neurones compared with astrocytes and endothelial cells. Von Willebrand factor (*VWF*), platelet and endothelial cell adhesion molecule 1 (*PECAM1*), and intercellular adhesion molecule 1 (*ICAM1*) were moderately expressed in endothelial cells compared with neurones and astrocytes. These findings suggest that the RNA isolated by immuno-LCM from toluidine blue-stained neurones and collagen IV-stained capillary endothelial cells represents an enriched neurone or endothelial cell population, respectively. Astrocyte-specific markers, *GFAP* and excitatory amino acid transporter 2 (*EAAT2*), were similarly expressed in neurones, astrocytes, and endothelial cells, reflecting the close proximity of astrocytes to the other cell types in the brain NVU (Additional file 6: Table S3).

### Differentially expressed transcripts in the brain NVU of cases with self-reported T2D

The transcription profiles of laser-captured neurones, astrocytes, and endothelial cells from the lateral temporal cortex of 6 cases with self-reported T2D and 5 age- and sex-matched controls were generated using Affymetrix Human Genome U133 Plus 2.0 GeneChip^Ⓡ^ Arrays. The gene expression data set is freely available at Gene Expression Omnibus (accession number GSE:161355). Between 18.5% and 65.7% of the probe set sequences were present across all three cell types (mean [range]): neurones (54.2% [38.5–65.7%]), astrocytes (43.4% [26.3–60.9%]), and endothelial cells (45.2% [18.5–61.5%]), respectively.

**Fig. 1:**
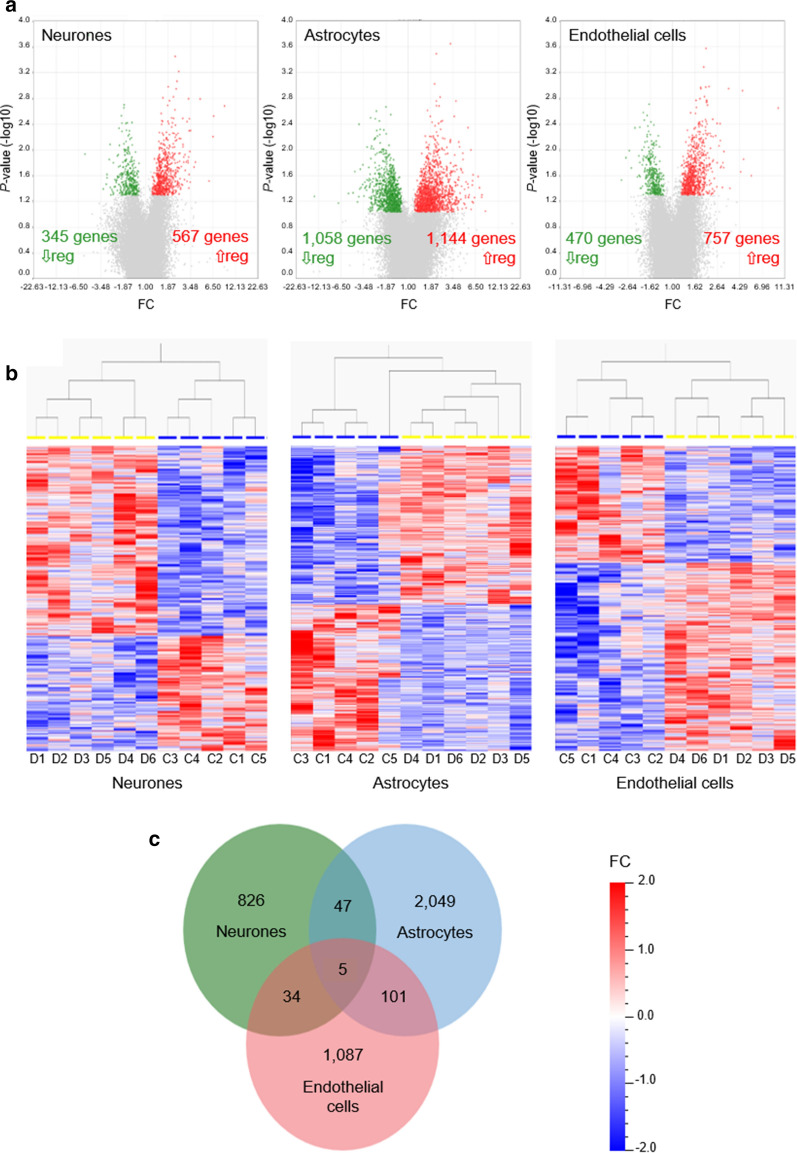
DEGs between T2D and control groups. **a** Volcano plots representing *P* value (−Log10 *P* value, vertical axis) and relative expression (FC, horizontal axis), **b** hierarchically clustered heat maps of DEGs in T2D [D] (yellow) versus controls [C] (blue), and **c** jVenn diagram comparing DEGs between different cell types of the NVU. Up-regulated (⇧ reg) genes are shown in red and down-regulated (⇩ reg) genes are shown in **a** green or **b** blue

Transcripts were considered significantly, differentially expressed if they had a minimum FC of plus or minus 1.2 and an unadjusted *P *< 0.05 (Student’s *t*-test). Nine hundred and twelve (567 up-regulated, 345 down-regulated), 2202 (1144 up-regulated, 1058 down-regulated), and 1227 (757 up-regulated, 470 down-regulated) significantly DEGs were identified in neurones, astrocytes, and endothelial cells, respectively (Fig. 1a). Principal component analysis and hierarchical clustering demonstrated clear separation between cases with self-reported T2D (highlighted in yellow) and controls (highlighted in blue) (Fig. 1b).

A Venn diagram comparing the lists of significantly DEGs (Fig. 1c) showed little overlap between the different cell types of the brain NVU at the specific gene level (in contrast to findings from the pathway analysis). Only 5 (0.1%) of the 4149 significantly DEGs were commonly dysregulated in neurones, astrocytes, and endothelial cells, namely DND microRNA-mediated repression inhibitor 1 (*DND1* probe set id: 57739_at) (neurones: FC, +1.33 [*P* = 0.014]; astrocytes: FC, +1.56 [*P *= 0.013]; endothelial cells: FC, +1.49 [*P *= 0.021]), leucine rich repeat containing 37A (*LRRC37A* probe set id: 239591_at) (neurones: FC, +2.09 [*P* = 0.034]; astrocytes: FC, +2.05 [*P *= 0.019]; endothelial cells: FC, +1.39 [*P *= 0.005]), NIMA related kinase 6 (*NEK6* probe set id: 223561_at) (neurones: FC, +1.58 [*P *= 0.027]; astrocytes: FC, +2.26 [*P *= 1.52E−05]; endothelial cells: +1.62 [*P *= 0.025]), oral cancer overexpressed protein 1 (*ORAOV1* probe set id: 243531_at) (neurones: FC, +1.85 [*P *= 0.049]; astrocytes: FC, +1.87 [*P *= 0.014]; endothelial cells: FC, +1.63 [*P *= 0.031]), and serpin family A member 3 (*SERPINA3* probe set id: 202376_at) (neurones: FC, +6.63 [*P *= 0.012]; astrocytes: FC, +4.10 [*P *= 0.018]; endothelial cells: FC, +3.50 [*P *= 0.009]). Forty-seven transcripts were commonly dysregulated in neurones and astrocytes (23 up-regulated, 6 down-regulated, 18 with opposite directions of change); 34 transcripts were commonly dysregulated in neurones and endothelial cells (17 up-regulated, 7 down-regulated, 10 with opposite directions of change); and 101 transcripts were commonly dysregulated in astrocytes and endothelial cells (72 up-regulated, 20 down-regulated, 9 with opposite directions of change).

IMPaLA pathway analysis identified 23, 65, and 34 pathways in the KEGG database that were significantly enriched in neurones, astrocytes, and endothelial cells, respectively (Wilcoxon test, unadjusted *P *< 0.05) (Additional file 4: Table S1).

### T2D-associated changes in the cortical neurone transcriptome

**Fig. 2:**
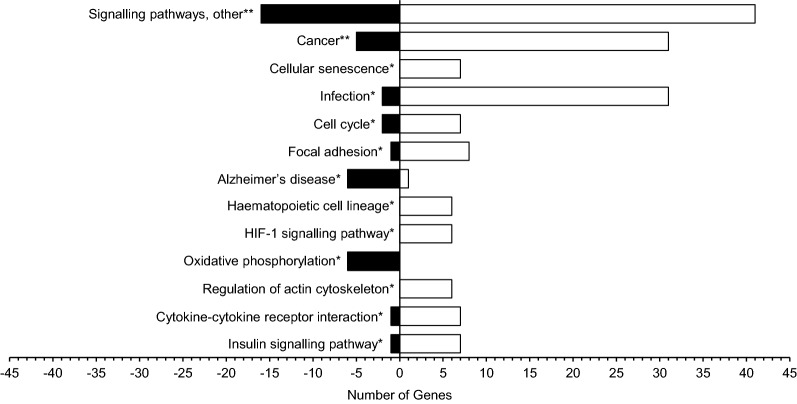
Neurone-enriched IMPaLA pathways in the KEGG database. Up-regulated genes are represented by the white bars and down-regulated genes are represented by the black bars. Signalling pathways, other includes PI3K-Akt, MAPK, Rap1, TNF, and retrograde endocannabinoid signalling pathways. *P < 0.05, **P < 0.01

Our further analysis focused on changes in pathways and functional groups, rather than individual DEGs. The neuronal transcriptomic profile indicated the significant dysregulation of several key signalling pathways associated with T2D, including phosphatidylinositol-3-kinase (PI3K)-protein kinase B (Akt) signalling (19 DEGs, *P *= 4.27E−03), cellular senescence (7 DEGs, *P *= 0.016), cell cycle (9 DEGs, *P *= 0.024), Alzheimer disease (7 DEGs, *P *= 0.031), HIF-1 signalling (6 DEGs, *P *= 0.031), tumour necrosis factor (TNF) signalling (6 DEGs, *P *= 0.031), oxidative phosphorylation (6 DEGs, *P *= 0.031), and insulin signalling (8 DEGs, *P *= 0.039) (Fig. 2).

Functional grouping identified marked dysregulation of multiple signalling cascades, including the activation of the PI3K-Akt signalling [forkhead box O 3 (*FOXO3* probe set id: 204131_s_at) (FC, +1.39; *P *= 0.020)], HIF-1 signalling [BCL2 apoptosis regulator (*BCL2* probe set id: 203684_s_at) (FC, +1.36; *P *= 0.028); cytochrome B-245 beta chain (*CYBB* probe set id: 203923_s_at) (FC, +2.74; *P *= 0.020)], TNF signalling [interleukin 6 (*IL6* probe set id: 205207_at) (FC, +1.43; *P *= 0.043)], and insulin signalling [glucose-6-phosphatase catalytic subunit 2 (*G6PC2* probe set id: 221453_at) (FC, +1.37; *P *= 0.032); phosphorylase kinase, alpha 1 (muscle) (*PHKA1* probe set id: 205450_at) (FC, +1.96; *P *= 4.91E−03); protein tyrosine phosphatase non-receptor type 1 (*PTPN1* probe set id: 217689_at) (FC, +1.70; *P *= 0.025); ribosomal protein S6 kinase B1 (*RPS6KB1* probe set id: 204171_at) (FC, +1.33; *P *= 0.026); sorbin and SH3 domain-containing protein 1 (*SORBS1* probe set id: 222513_s_at) (FC, +1.51; *P *= 2.85E−03)] pathways (Fig. 2).

Further interrogation of the cortical neurone transcriptome identified dysregulation of genes implicated in the cell cycle and cellular senescence, including checkpoint kinase 1 (*CHEK1* probe set id: 205394_at) (FC, +1.44; *P *= 0.025), cyclin-dependent kinase 1 (*CDK1* probe set id: 210559_s_at) (FC, +1.52; *P *= 0.022), cell division cycle 14A (*CDC14A* probe set id: 210440_s_at) (FC, +2.13; *P *= 0.015), stromal antigen 1 (*STAG1* probe set id: 202294_at) (FC, +1.44; *P *= 0.037), and transcription factors Dp-1 (*TFDP1* probe set 242939_at) (FC, −1.30; *P *= 0.047), and Dp-2 (*TFDP2* probe set id: 226157_at) (FC, +1.34; *P *= 1.84E−03).

Pathway analysis also identified dysregulation of AD-related genes, including amyloid beta (A4) precursor protein-binding, family B, member 1 (*APBB1* probe set id: 202652_at) (FC, −1.64; *P *= 0.026) and membrane metalloendopeptidase (neprilysin) (*MME* probe set id: 203434_s_at) (FC, +1.37; *P *= 0.049). Specifically, there was down-regulation of mitochondrial respiratory electron transport chain-associated transcripts, including cytochrome C oxidase subunit 5B (*COX5B* probe set id: 213735_s_at) (FC, −1.17; *P *= 0.095) and NADH:ubiquinone oxidoreductase subunits A1 (*NDUFA1* probe set id: 202298_at) (FC, −1.46; *P *= 0.014), A8 (*NDUFA8* probe set id: 218160_at) (FC, −1.56; *P *=0.012), B3 (*NDUFB3* probe set id: 203371_s_at) (FC, −1.71; *P *= 0.029), and B6 (*NDUFB6* probe set id: 1559042_at) (FC, −1.50; *P *= 0.041).

### Concurrent changes in the brain NVU: astrocyte and endothelial cell transcriptomes

**Fig. 3:**
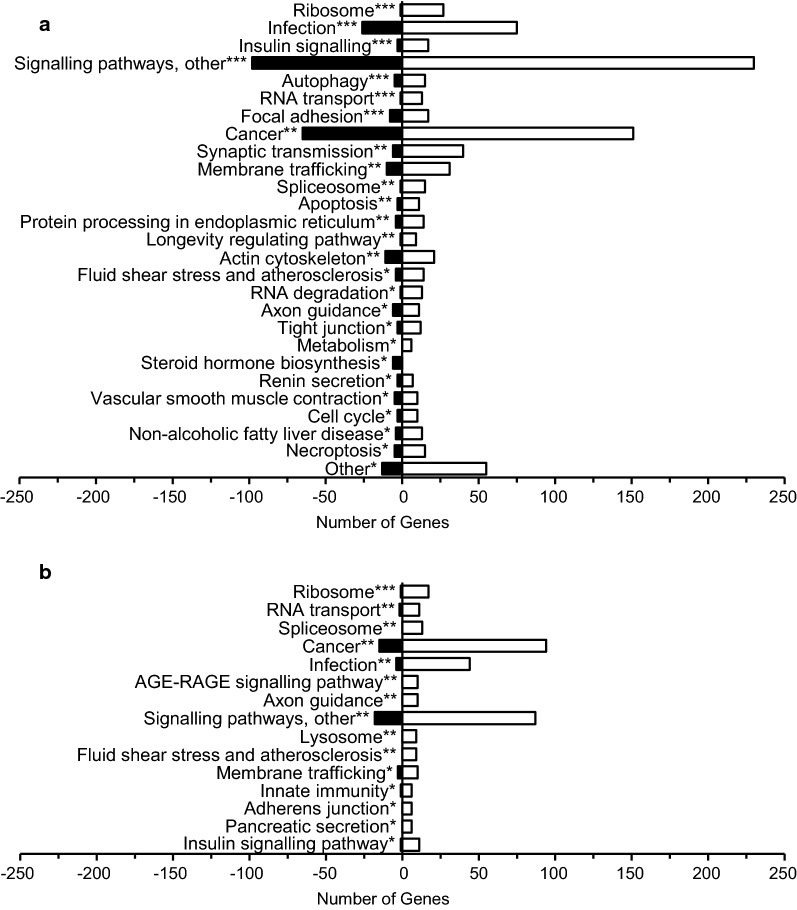
**a** Astrocyte- and **b** endothelial cell-enriched IMPaLA pathways in the KEGG database. Up-regulated genes are represented by the white bars and down-regulated genes are represented by the black bars. Signalling pathways, other includes **a** Sphingolipid, oxytocin, Rap1, neurotrophin, ErbB, phosphatidylinositol, MAPK, chemokine, T cell receptor, GnRH, Ras, AMPK, mTOR, PI3K-Akt, oestrogen, Wnt, and p53 and **b** PI3K-Akt, oxytocin, phospholipase D, GnRH, Rap1, MAPK, thyroid hormone, cGMP-PKG, and Wnt signalling pathways. *P < 0.05, **P < 0.01, ***P < 0.001

To set the neuronal changes in the context of the brain NVU, we expanded our analysis to examine the concurrent pathway alterations in the astrocyte and endothelial cell transcriptomes. Significantly enriched KEGG pathways in the astrocyte transcriptome included insulin signalling (20 DEGs, *P *= 4.18E−04), autophagy (20 DEGs, *P *= 8.51E−04), apoptosis (14 DEGs, *P *= 5.23E−03), fluid shear stress and atherosclerosis (18 DEGs, *P *= 0.013), chemokine signalling (17 DEGs, *P *= 0.015), p53 signalling (11 DEGs, *P *= 0.042), cell cycle (13 DEGs, *P *= 0.043), Non-alcoholic fatty liver disease (NAFLD, 17 DEGs, *P *= 0.047), and necroptosis (15 DEGs, *P *= 0.047) (summarised in Fig. 3a; see Additional file 4: Table S1 for full listing). Significantly enriched KEGG pathways in the endothelial cell transcriptome also included insulin signalling (12 DEGs, *P *= 0.034) and fluid shear stress and atherosclerosis (9 DEGs, *P *= 9.03E−03), phospholipase D signalling (8 DEGs, *P *= 0.016) and AGE-RAGE signalling in diabetic complications (10 DEGs, *P *= 5.89E−03) (Fig. 3b).

### Validation of key neuronal targets

**Fig. 4:**
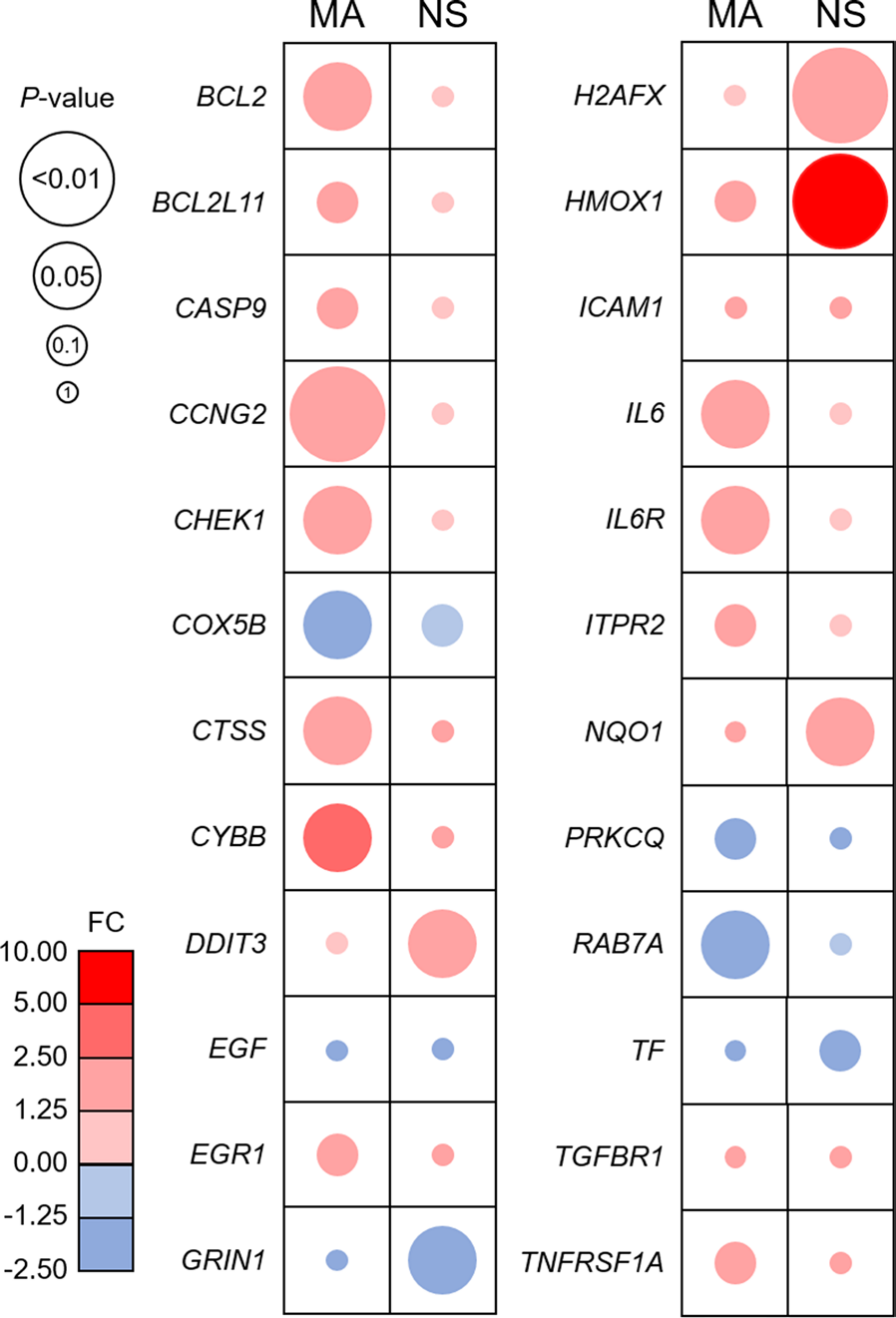
Validation of neurone microarray data using the NanoString nCounter Platform. Up-regulated genes are represented by the red circles and down-regulated genes are represented by the blue circles. The size of the circle indicates the significance of the *P* value. Colour intensity denotes the magnitude of the FC, fold-change; MA, microarray; NS, NanoString

Neuronal expression of selected candidate genes identified through WGCNA was validated in the same cohort as the microarray, using the NanoString nCounter platform. The same direction of change was confirmed for 63 (91.3%) of the 69 genes in the custom C6959X1 CodeSet. Transcriptional alterations associated with T2D included the increased expression of DNA damage inducible transcript 3 (*DDIT3* probe set id: 209383_at) (FC, +2.03; *P* = 0.036), H2A histone family, member X (*H2AFX* probe set id: 205436_s_at) (FC, +1.71; *P* = 2.80E−03), heme oxygenase 1 (*HMOX1* probe set id: 203665_at) (FC, +8.38; *P *= 7.86E−03), and NAD(P)H quinone dehydrogenase 1 (*NQO1* probe set id: 201468_s_at) (FC, +2.26; *P *= 0.026), and the decreased expression of *COX5B* (probe set id: 213735_s_at) (FC, –1.17; *P *= 0.095), glutamate ionotropic receptor NMDA type subunit 1 (*GRIN1* probe set id: 210781_x_at) (FC, –1.32; *P *= 0.024), and transferrin (*TF* probe set id: 220109_at) (FC, –1.59; *P *= 0.092) (Fig. 4).

**Fig. 5:**
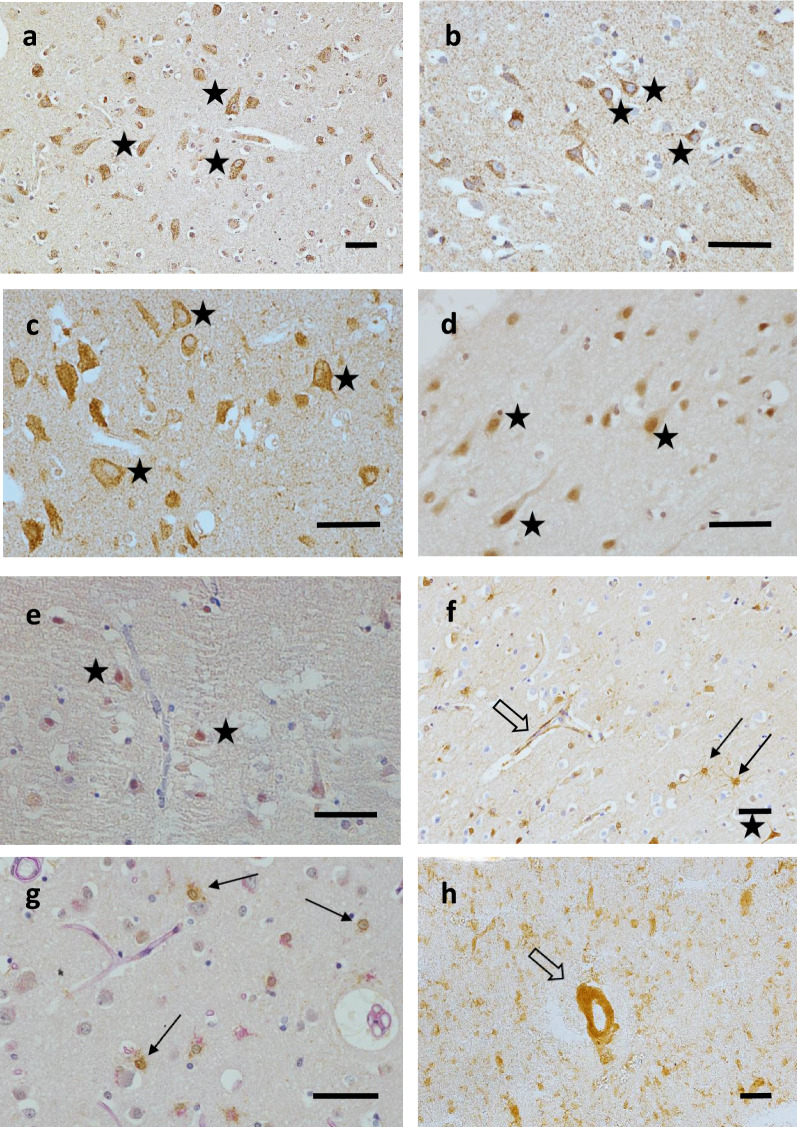
Immunohistochemical staining. **a** COX5b, Case D4; **b** NDUFb6, Case D6; **c** TGFβ1, Case D5; **d** FOXO3a, Case D5; **e** p53, Control C4; **f** AGE, Case D6; **g** γH2AX, Case D6; **h** IGF-1Rβ, Case D3. immunoreactivity in neurones (stars), astrocytes (arrows), and endothelial cells (open arrows) in lateral temporal cortex from CFAS cases in the microarray cohort. Magnification bars 50 μm

The neuronal expression and cellular localisation of proteins encoded by selected candidate genes, namely COX5b, NDUFb6, transforming growth factor beta 1 (TGFβ1), FOXO3a, and tumour protein p53 (p53), were confirmed by immunohistochemistry (Fig. 5a–e). Further immunohistochemical analysis revealed positive staining for AGEs in all three cell types of the brain NVU (neurones, astrocytes, and endothelial cells), γH2AX in astrocytes, and insulin-like growth factor 1 receptor beta (IGF-1Rβ) in endothelial cells (Fig. 5f–h).

## Discussion

### Key findings

T2D is a major modifiable risk factor for dementia. Whilst some of this risk may be mediated via its effects on classical dementia-associated neuropathologies, particularly vascular disease, T2D may directly affect cells of the brain. To address this question, we examined changes in the neuronal transcriptome in neocortex associated with self-reported T2D using microarray-based gene expression analysis of RNA isolated from laser-captured neurones. Additionally we examined the transcriptomes of astrocytes and endothelial cells to set the neuronal changes in the context of the broader NVU and to determine whether the three cell types were similarly impacted by T2D. Laser-capture microdissection produces cell-type enriched, but not pure populations [53, 63]. This was reflected in the astrocytic transcripts identified in neuronal and endothelial populations. We did not specifically examine pericytes, but given the resolution of laser capture microdissection these will also be represented in the “endothelial samples”, along with astrocytic processes so that endothelial enriched samples reflect the more complex cells of the microvasculature [40]. Methods such as RNA in situ hybridisation may be of value in future work to obtain clearer cell-type specific expression of candidate genes at higher resolution. This would allow separation of pericytic and endothelial signals, for example, but our primary focus in this study was on the neuronal changes.

The cases used were selected to have low levels of potentially confounding neuropathology. Gene expression analysis of the neuronal transcriptome showed separation of T2D from control cases with 912 DEGs. Pathway analysis revealed changes in key neuronal pathways that are relevant to potential neuronal dysfunction in dementia, including signalling, cell cycle and senescence, and AD-related pathways. Gene expression changes were validated using NanoString as a second independent method. Key pathway alterations were also identified in astrocytes and endothelial cells, including inflammation pathways, insulin signalling and AGE-RAGE signalling pathways. Although there was little overlap in the DEGs between the three cell types at the individual gene level, some of the signalling pathway changes were shared, particularly insulin signalling which suggests that insulin signalling is widely dysregulated in the NVU.

### Changes in the neuronal transcriptome

Identifying patterns of altered genes forming cellular pathways can potentially provide a more physiologically relevant picture of cellular alterations and be less susceptible to false discovery than individual genes. Using this approach we have previously identified pathogenically relevant pathway alterations in the CFAS cohort in relation to oxidative DNA damage, astrocyte changes with AD neuropathology progression and in white matter lesions [50–52]. We therefore focused on changes in cellular pathways and functional groups. T2D can affect a variety of metabolic processes that can affect cell function, including insulin and other signalling pathways, glucose metabolism and mitochondrial function, processes the may contribute to neuronal dysfunction [11]. Using pathway analysis, we have herein identified changes in signalling pathways, cell cycle and senescence, and AD-related transcripts in neurones in association with T2D.

Signalling pathway alterations included changes in insulin, PI3K-Akt, HIF-1 and TNF signalling pathways suggesting that neuronal signalling is dysregulated in T2D. Alterations in neuronal insulin signalling have been implicated in the pathogenesis of AD and may affect the downstream PI3K-Akt pathway. There may be several mechanisms for reduced activation of the insulin signalling pathway in AD neurones, including the binding of Aβ to the insulin receptor and changes in the phosphorylation state of insulin receptor substrate 1 [37, 57, 70]. Reduced expression of mRNAs for insulin (and other) signalling pathway molecules have been identified in astrocytes in association with the progression of AD neuropathological change [52]. Insulin resistance in neurones experimentally may also contribute to AD molecular pathology [10, 68]. We also identified reduced expression of insulin signalling pathway genes in association with a DNA damage response in neurones in human autopsy tissue [51]. Impaired neuronal insulin signalling therefore appears to be an important common mechanism in neurones. Results from *this* study suggest that neuronal insulin signalling is also affected in T2D, and most of the pathway components were up-regulated, which might imply a compensatory response to impaired insulin signalling.

Genes in the cell cycle and senescence pathways were also altered. Senescent cells show loss of function, may propagate damage through the senescence-associated secretory pathway and have been implicated in AD [64]. Senescent cells are also a potential therapeutic target, with the advent of senolytics [26, 30, 41]. Whether this may lead to neuronal senescence in T2D is therefore a pressing question.

Pathway analysis also revealed alteration in AD-related transcripts. These include Aβ-related transcripts and neprilysin, which is the dominant Aβ peptide-degrading enzyme in the brain [17]. Genes in this pathway also suggest down-regulation of mitochondrial genes, which may imply mitochondrial dysfunction. Mitochondrial dysfunction has been implicated in neurodegeneration including AD, as well as in diabetes and obesity [45]. Alterations in these pathways provide a potential point of intersection between T2D and AD neuropathology.

### Cell pathway alterations in astrocytes and endothelial cells

We identified gene expression and cell pathway alterations in astrocytes and endothelial cells that suggest they are also involved in T2D-related brain injury. There is increasing evidence for the importance of astrocytes in dementia and neurodegenerative disorders [13, 20] whilst endothelial cells are the interface between the brain and blood, and would be expected to be affected by T2D. In common with changes in neurones, pathway analysis also revealed changes in insulin signalling in astrocytes and endothelial cells, suggesting that insulin signalling dysregulation of the various cellular components of the NVU is a feature of T2D. These are changes that might be expected in diabetes providing further support for the relevance of pathway changes found. Insulin signalling impairment is a feature of neurones in AD, but this pathway is also functional in astrocytes [19] and changes in this pathway have also been found in astrocytes with AD neuropathology progression [52], suggesting that these cells and possibly also cerebral endothelial cells may be affected by IR in T2D. The NAFLD pathway is also altered in astrocytes. Non-alcoholic fatty liver disease, recently renamed metabolic-associated fatty liver disease, is a feature of T2D and metabolic syndrome [31].The NAFLD pathway contains genes relevant to insulin signalling and mitochondrial function (Additional file 4: Table S1), further suggesting metabolic and signalling dysregulation in astrocytes and links to peripheral dysmetabolism. Alterations in autophagy, apoptosis, p53 signalling and cell cycle, suggest astrocyte injury and changes to proteostasis. Astrocytes are an important source of glutamate for neurons as part of the tripartite synapse [20] and changes to genes involved in glutamate reuptake and cycling may imply a protective response from excitotoxicity. Endothelial cells showed alterations in the AGE-RAGE signalling pathway, which has pleiotropic cellular effects and has been implicated in neuroinflammation and neurodegeneration [14]. AGE formation can result from abnormal glycation and we recently showed that it increases with AD neuropathological change, although it did not associate with dementia and the relationship of brain AGE formation to T2D is yet to be defined [9]. AGE formation may therefore be one mechanism of endothelial damage in T2D. Changes in the cellular adherence pathway in endothelial cells could be a response to maintain blood–brain barrier integrity. Overall, these changes suggest that T2D affects astrocytes and endothelial cells as well as neurons. These changes may contribute to NVU dysfunction, but may also reveal compensatory mechanisms.

### Study limitations

The CFAS neuropathology cohort is population-representative. However, this study used a case–control design nested within the larger population study. Cases were defined according to self-reported T2D status. However, defining T2D for retrospective studies and quantifying the severity and cumulative exposure to metabolic dysregulation are problematic [7]. Conversely, whilst our control group did not have self-reported T2D, we cannot exclude the possibility of subclinical T2D or undiagnosed metabolic syndrome. Such difficulties would require a prospective case–control cohort to resolve, with longitudinal follow-up to provide a more integrated measure of in-life T2D/MS over a time course. Such a cohort would be valuable to further understand the effects of T2D/MS on the brain and for validation of the changes we have identified in a separate cohort. Given potential interactions between T2D/MS and AD, it would also be of value to compare changes associated with each of these individual pathologies and cases with combined pathology to determine the effects of interaction on the NVU.

A further limitation of this study was the number of cases available, a limitation imposed by the number of cases in the CFAS neuropathology cohort with self-reported T2D, but with good quality RNA and without confounding pathologies. Whilst the number used here has detected significant changes in gene expression and in key cellular pathways, and similar numbers have generated robust results in previous studies [44, 59], some studies suggest that a larger number of cases is required to reliably detect all DEGs. For example, Schurch et al. [48] reported that for RNA-sequencing gene expression studies, group sizes should be 12 or more to detect all DEGs. With a smaller number of cases in this study, it is possible that some significantly DEGs were not detected.

## Conclusions

Most investigations into the effects of T2D on the brain have focused on interactions with vascular disease and AD. This study, using two methods to assess transcriptomic changes, identified pathogenically relevant pathway changes in cortical neurones in association with T2D, with changes in astrocytes and endothelial cells suggesting wider involvement of the NVU. A transcriptomic approach, as used here, is a hypothesis generating approach that requires further investigation in separate cohorts and experimental systems. However, these changes offer potential new avenues for mechanistic, biomarker and therapeutic studies. Neuronal gene expression and pathway alterations suggest dysregulation of signalling, cell injury, neuroinflammation and metabolism suggesting potential mechanisms by which T2D may directly affect the brain, producing a diabetic dementia that is independent of but potentially interacting with vascular and AD neuropathology.

## Supplementary Information

**Additional file 1 Fig. S1** Diabetes and Dementia Network. WGCNA of IMPaLA-generated lists of DEGs enriched in insulin signalling, AGE-RAGE signalling in diabetic complications, and Alzheimer disease pathways in the KEGG database. Hub genes included in the NanoString panel are highlighted in yellow. Targets which were validated by immunohistochemistry are indicated by the asterisks. Two gene products are linked in red, if they participated in the same reaction within a pathway. The strengths of relationships are represented by the intensity of the colour and the thickness of the interconnecting lines. Created using the GeneMANIA plugin for Cytoscape version 3.7.2

**Additional file 2 Fig. S2** Diabetic Complications Network. WGCNA of IMPaLA-generated lists of DEGs enriched in AGE-RAGE signalling in diabetic complications, chemokine signalling, HIF-1 signalling, fluid shear stress and atherosclerosis, and NAFLD pathways in the KEGG database. Hub genes included in the NanoString panel are highlighted in yellow. Targets which were validated by immunohistochemistry are indicated by the asterisks. Two gene products are linked in red, if they participated in the same reaction within a pathway. The strengths of relationships are represented by the intensity of the colour and the thickness of the interconnecting lines. Created using the GeneMANIA plugin for Cytoscape version 3.7.2

**Additional file 3 Fig. S3** (**A**) DNA Damage Response and (**B**) Autophagy Networks. WGCNA of IMPaLA-generated lists of DEGs enriched in (**A**) cell cycle, cellular senescence, p53 signalling, apoptosis, and necroptosis and (**B**) autophagy pathways in the KEGG database. Hub genes included in the NanoString panel are highlighted in yellow. Targets which were validated by immunohistochemistry are indicated by the asterisks. Two gene products are linked in red, if they participated in the same reaction within a pathway. The strengths of relationships are represented by the intensity of the colour and the thickness of the interconnecting lines. Created using the GeneMANIA plugin for Cytoscape version 3.7.2

**Additional file 4 Table S1** IMPaLA-Enriched Pathways in the KEGG Database. #, number; IMPaLA, Integrated Molecular Pathway Level Analysis; KEGG, Kyoto Encyclopaedia of Genes and Genomes

**Additional file 5 Table S2** NanoString nCounter Custom CodeSet: C6959X1. *P < 0.05, **P < 0.01. #, number; CTRL, control; CUS, custom; FC, fold-change; HK, housekeeping; Hs, *Homo sapiens*; mRNA, messenger RNA; NEG, negative; POS, positive; *sp.*, species

**Additional file 6 Table S3** Neurone-, Astrocyte-, and Endothelial Cell-Specific Transcripts. RMA, robust multi-array average

## Data Availability

Gene expression data is available in the Gene Expression Omnibus.
