# Supplementary figures and images for: Type 2 diabetes mellitus-associated transcriptome alterations in cortical neurones and associated neurovascular unit cells in the ageing brain
Source: Acta Neuropathol Commun. 2021 Jan 6;9:5. doi: 10.1186/s40478-020-01109-y (PMC7788898; doi:10.1186/s40478-020-01109-y)

## Slide 1
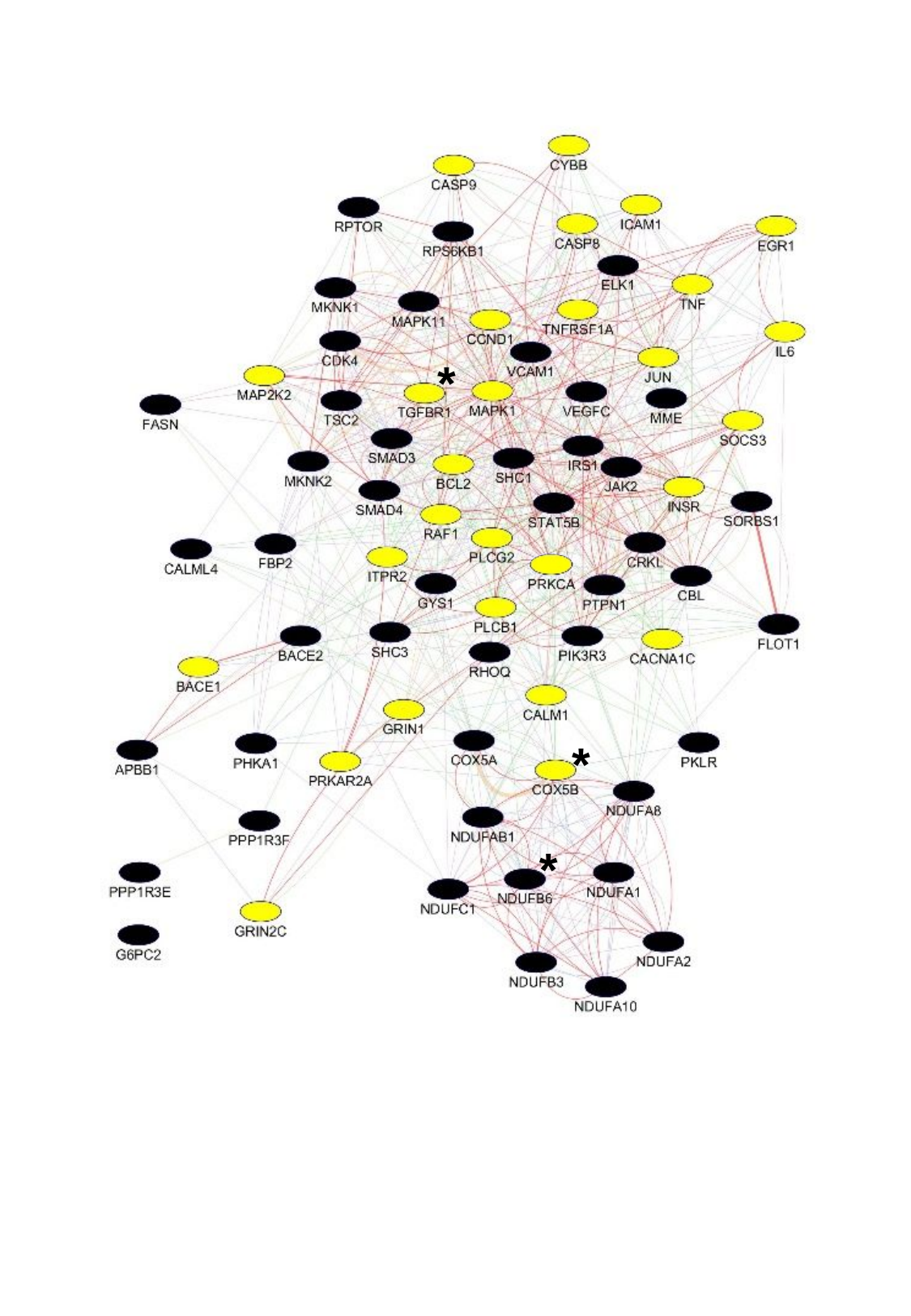

*
*
*

Supplement: Supplementary file 1 — Additional file 1 Fig. S1 Diabetes and Dementia Network. WGCNA of IMPaLA-generated lists of DEGs enriched in insulin signalling, AGE-RAGE signalling in diabetic complications, and Alzheimer disease pathways in the KEGG database. Hub genes included in the NanoString panel are highlighted in yellow. Targets which were validated by immunohistochemistry are indicated by the asterisks. Two gene products are linked in red, if they participated in the same reaction within a pathway. The strengths of relationships are represented by the intensity of the colour and the thickness of the interconnecting lines. Created using the GeneMANIA plugin for Cytoscape version 3.7.2 [file 40478_2020_1109_MOESM1_ESM.pptx]

## Slide 1
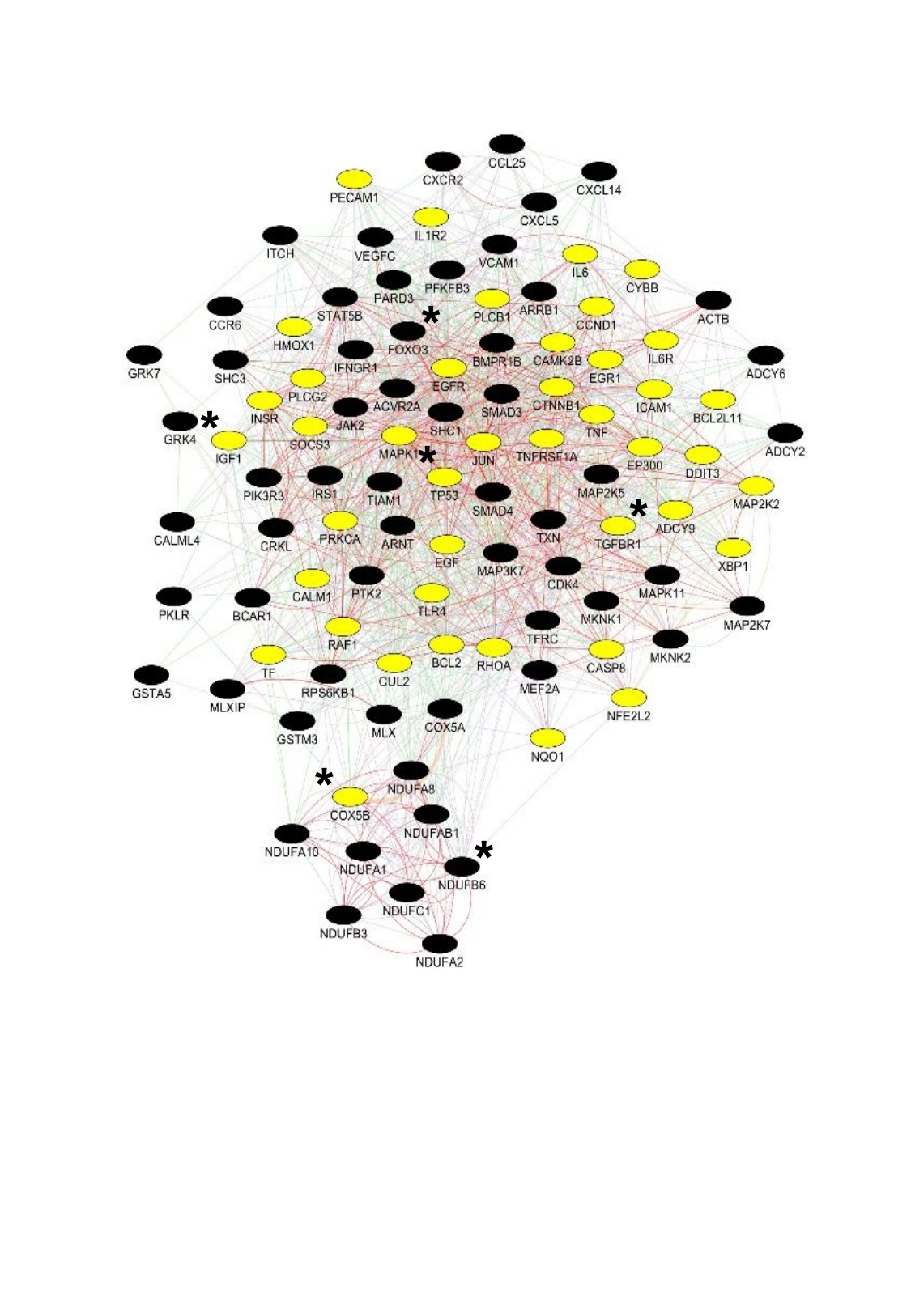

*
*
*
*
*
*

Supplement: Supplementary file 2 — Additional file 2 Fig. S2 Diabetic Complications Network. WGCNA of IMPaLA-generated lists of DEGs enriched in AGE-RAGE signalling in diabetic complications, chemokine signalling, HIF-1 signalling, fluid shear stress and atherosclerosis, and NAFLD pathways in the KEGG database. Hub genes included in the NanoString panel are highlighted in yellow. Targets which were validated by immunohistochemistry are indicated by the asterisks. Two gene products are linked in red, if they participated in the same reaction within a pathway. The strengths of relationships are represented by the intensity of the colour and the thickness of the interconnecting lines. Created using the GeneMANIA plugin for Cytoscape version 3.7.2 [file 40478_2020_1109_MOESM2_ESM.pptx]

## Slide 1
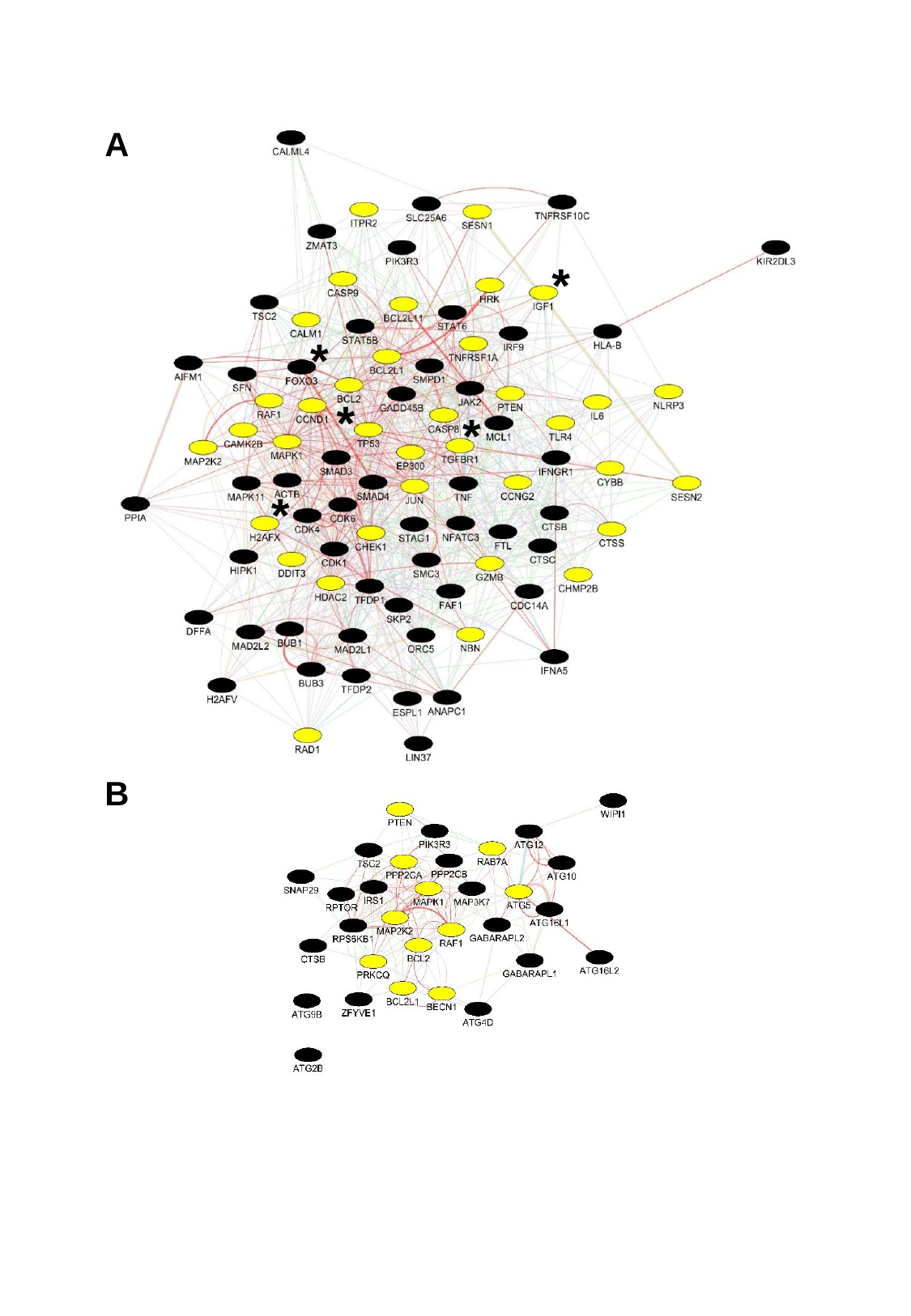

*
*
*
*
*
B
A

Supplement: Supplementary file 3 — Additional file 3 Fig. S3 (A) DNA Damage Response and (B) Autophagy Networks. WGCNA of IMPaLA-generated lists of DEGs enriched in (A) cell cycle, cellular senescence, p53 signalling, apoptosis, and necroptosis and (B) autophagy pathways in the KEGG database. Hub genes included in the NanoString panel are highlighted in yellow. Targets which were validated by immunohistochemistry are indicated by the asterisks. Two gene products are linked in red, if they participated in the same reaction within a pathway. The strengths of relationships are represented by the intensity of the colour and the thickness of the interconnecting lines. Created using the GeneMANIA plugin for Cytoscape version 3.7.2 [file 40478_2020_1109_MOESM3_ESM.pptx]
